# Feeling Touch *Through* Glass: A Modified Rubber Hand Paradigm

**DOI:** 10.1177/2041669517731114

**Published:** 2017-09-12

**Authors:** Rebekah C. White, Jiexin Li, David Shacklette

**Affiliations:** Department of Experimental Psychology, University of Oxford, Oxford, UK; Pembroke College, University of Oxford, UK; Zhejiang University, Hangzhou, China; Pembroke College, University of Oxford, UK; Illinois Wesleyan University, Bloomington, USA

**Keywords:** rubber hand illusion, predictive signals, tactile congruency, body representation

## Abstract

A variation on the rubber hand paradigm creates a striking illusion in which it seems to the participant that she or he is feeling touch through glass. This illusion provides insight about how individuals make use of predictive signals for integrating vision and touch.

Over the past two decades, there has been an exponential increase in the number of experiments employing the rubber hand paradigm to investigate body awareness and representation. In the traditional version of the paradigm (Botvinick & Cohen, 1998), a prosthetic left hand is positioned on a table top in full view of the participant, and the participant’s left hand is hidden from view. (Note that the procedure can be adapted to a right hand, but for the sake of simplicity, we describe the methods as they apply to a left prosthetic hand and the participant’s left hand.) When the examiner administers *synchronous* touch to the two hands, it may seem to the participant that she or he is feeling touch at the location of the prosthetic hand (visual capture of touch), that the felt touch is caused by the paintbrush touching the prosthetic hand (illusion of causation), and that the prosthetic hand is the participant’s hand (illusion of ownership). This experience is referred to as the rubber hand illusion (RHI).

In a tutorial on experimental methods and design, we experimented with the set-up of the rubber hand paradigm and discovered that the RHI is resistant to a surprising manipulation. We made use of the furniture that was available in the tutorial room. The examiner and participant were seated on opposite sides of a glass-topped coffee table. The viewed prosthetic hand was positioned just *below* the glass and the participant’s own left hand was positioned *on top* of the glass and hidden from view by way of a visual divider (Figure 1). Using one paintbrush, the examiner stroked the glass surface above the prosthetic hand, and using a second paintbrush, the examiner administered synchronous strokes to the participant’s hidden left hand. Despite the viewed prosthetic hand being positioned below a sheet of glass, within 60 seconds, each of us experienced a compelling RHI.

**Figure 1.**
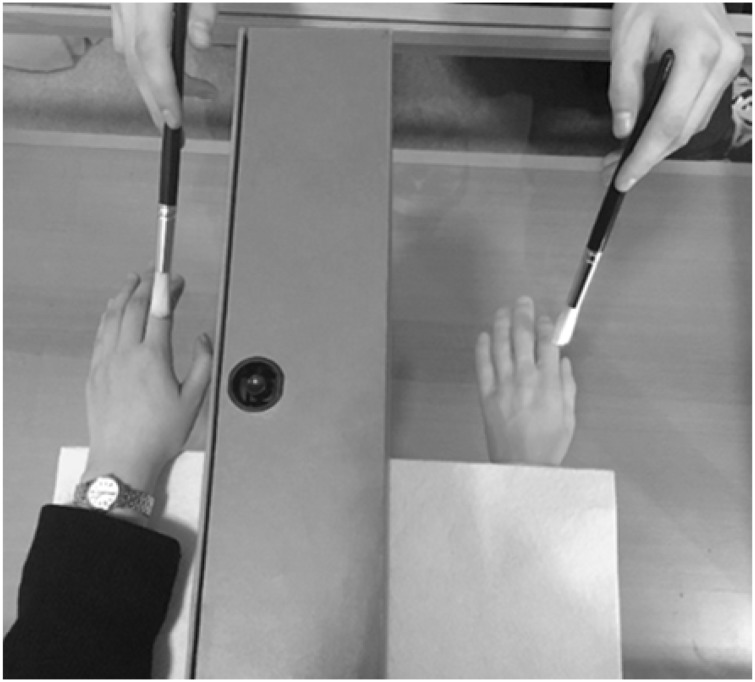
The experimental set-up. The participant’s left hand was positioned on the glass table top to the left of the visual divider. The prosthetic left hand was positioned beneath the glass table top to the right of the visual divider. The participant was seated so that the visual divider was in line with the participant’s left shoulder, thereby preventing the participant from seeing her or his own left hand. Note that a piece of cloth, placed on top of the table, concealed the arm of the prosthetic hand.

Subsequently, we tested 12 new participants (10 women and 2 men; 18–60 years, *M* = 36 years) who were naïve to the RHI. There were three 60-second trials: (a) our novel glass rubber hand paradigm with synchronous stimulation, (b) the traditional rubber hand paradigm with synchronous stimulation, and (c) the glass rubber hand paradigm with asynchronous stimulation. Experience of the RHI was assessed using Botvinick and Cohen’s (1998) nine-item questionnaire (Figure 2). Three participants did not experience the RHI on any trial. The remaining nine participants experienced a compelling illusion on *both* of the synchronous trials. Seven of the nine participants gave identical agreement ratings across the two trials, suggesting that the glass and traditional paradigms elicit illusions of comparable strength.

**Figure 2.**
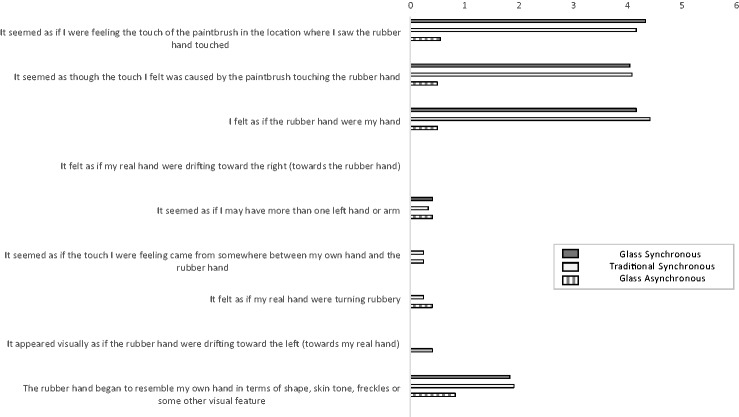
Mean agreement ratings for Botvinick and Cohen’s (1998) questionnaire. Statements 1 to 3 in the figure assessed the illusion (visual capture, causation, ownership) and Statements 4 to 9 were included to control for suggestibility. (Note that the order of statements was randomized.) Agreement ratings were provided on a 7-point scale ranging from 0 (*not at all*) to 6 (*very strongly agree*).

How does the experience of our glass RHI sit with the existing literature? Schütz-Bosbach, Tausche, and Weiss (2009) found that the RHI was not diminished by tactile incongruence: The viewed prosthetic hand received touch from a *rough* stimulus, while the participant’s hidden hand received touch from a *soft* stimulus (and vice versa: for similar findings from the nonvisual RHI, see White, Aimola Davies, Halleen, & Davies, 2010). In contrast, Ward, Mensah, and Jünemann (2015) found that a more dramatic tactile incongruence abolished the RHI: The viewed prosthetic hand received touch from a *hard* stimulus, while the participant’s hidden hand received touch from a *soft* stimulus. Drawing on the results from the two studies, Ward et al. suggested that participants use visual and somatosensory signals to make predictions about what touch should feel and look like, and with a sufficiently large discrepancy, the RHI is not elicited. They suggested that incongruency is detected in the somatosensory system, rather than via a more conceptual process based on the participant’s understanding of stimulus properties. Thus, the hard–soft difference is more dissimilar at the perceptual level (but perhaps not the conceptual level) than the rough–soft difference. Aimola Davies and White (2013) found that the RHI was not elicited with a no-touch rubber hand paradigm in which the viewed prosthetic hand was stroked with a paintbrush (touch) while the participant’s hidden hand did not receive any stimulation (no touch). Ward et al. suggested that the absence of the RHI with this set-up was due to there being “gross incongruity between what is predicted from vision and what is actually felt” (p. 1207). And yet, with our glass rubber hand paradigm, there is also gross incongruity between what is predicted from vision and what is actually felt, insofar as the viewed prosthetic hand does not receive *any* stimulation due to it being positioned beneath a sheet of glass (no touch), whereas the participant’s hidden hand does receive stimulation from a paintbrush (touch). Despite this, participants experience a compelling RHI.

This leads us to make a proposal about how the participant (or comparator system) makes use of visual and tactile signals. In making predictions about what touch should feel like, the participant considers what the visual stimulus would feel like if applied to her or his own hand (i.e., brushstrokes), rather than what the visual stimulus must feel like as it is applied to the viewed hand (i.e., no sensation due to the presence of an intermediary sheet of glass). And likewise, in making predictions what touch should look like, the participant considers what the administering tactile stimulus would look like (i.e., a paintbrush), rather than what the administered tactile stimulation would look like (i.e., a paintbrush administering strokes directly to the hand).

The findings from our glass rubber hand paradigm feed into the debate about how top-down information (e.g., knowledge and expectations) affects the RHI. Although we know that humans cannot feel touch through glass, this knowledge is insufficient to abolish the RHI. We might consider that the paradigm elicits an illusion on top of an illusion. As with the traditional rubber hand paradigm, the participant disregards proprioceptive information to experience touch on a viewed hand, and in addition, the participant perceives that she or he is feeling this touch through a sheet of glass. We hope that the glass RHI will offer a new window through which to interpret past findings. As but one example, many studies have shown that the RHI is not elicited when the viewed prosthetic hand is replaced with a nonhand object (e.g., a block of wood: Tsakiris, Carpenter, James, & Fotopoulou, 2010). One might question whether this is due to higher order beliefs about the types of objects that could plausibly feel touch, but our glass RHI argues against this interpretation: Although humans cannot feel touch through glass, the RHI is elicited with this novel manipulation.
